# Effect of Low Glycemic Load Diet on Glycated Hemoglobin (HbA1c) in Poorly-Controlled Diabetes Patients

**DOI:** 10.5539/gjhs.v4n1p211

**Published:** 2012-01-01

**Authors:** Amir Ziaee, Ahmad Afaghi, Majied Sarreshtehdari

**Affiliations:** Qazvin Metabolic Diseases Research Center Qazvin University of Medical Sciences; Assistant professor of Qazvin University of Medical Science Metabolic Disease Research Center, Qazvin, Iran E-mail: aafa2000@gmail.com; Qazvin Metabolic Diseases Research Center Qazvin University of Medical Sciences

**Keywords:** Poorly-controlled diabetes, Glycemic load, Glycated hemoglobin

## Abstract

Different carbohydrate diets have been administrated to diabetic patients to evaluate the glycemic response, while Poor-controlled diabetes is increasing world wide. To investigate the role of an alternative carbohydrate diet on glycemic control, we explored the effect of a low glycemic load (Low GL)-high fat diet on glycemic response and also glycated hemoglobin (HbA1c) of poor-controlled diabetes patients. Hundred poorly-controlled diabetes patients, HbA1c > 8, age 52.8 ± 4.5 y, were administrated a low GL diet, GL = 67 (Energy 1800 kcal; total fat 36%; fat derived from olive oil and nuts 15%; carbohydrate 42%; protein 22%) for 10 weeks. Patients did their routine life style program during intervention. Fasting blood glucose and HbA1c before and after intervention with significant reduction were: 169 ± 17, 141 ± 12; 8.85% (73 mmol/mol) ± 0.22%, and 7.81% (62 mmol/mol) ± 0.27%; respectively (P < 0.001). Mean fasting blood glucose reduced by 28.1 ± 12.5 and HbA1c by 1.1% (11 mmol/mol) ± 0.3% (P=0.001). There was positive moderate correlation between HbA1c concentration before intervention and FBS reduction after intervention (P < 0.001, at 0.01 level, R =0.52), and strong positive correlation between FBS before intervention and FBS reduction (P < 0.001, at 0.01 level, R = 0.70). This study demonstrated that our alternative low glycemic load diet can be effective in glycemic control.

## 1. Introduction

Poorly-controlled diabetes that is characterized with increased glycated hemoglobin (HbA1c) > 8% (64 mmol/mol) (Mahan and Escott-Stump, 2007) is increasing world wide, especially in North America and Europe which resulted in an increasing prevalence of disease associated with poor glycemic control (Livesey and Tagami, 2009). Different interventions to lower the glycemic response to carbohydrate foods have been introduced. Theses approaches included: Diets containing 50-60% calories from carbohydrates (Arora and McFarlance, 2005), consumption of soluble fiber, non-soluble fiber, low viscosity fiber (resistant maltodextrin) (Livesey and Tagami, 2009), and administration of low glycemic load diet (100 g) (glucose equivalents per day) without elevating fat intake (Livesey and Tagami, 2009). High carbohydrate intake recommended in diabetes, resulting in suboptimal glycemic control and lipoprotein profile, gradually increasing insulin and/or oral hypoglycemic medication requirement and eventually weight gain (Arora and McFarlance, 2005, 2:16). Several studies have demonstrated that viscous soluble fibers suppress the glycemic response to carbohydrate foods, (Garcia *et al.*, 2007; Livesey *et al.*, 2008), and beneficial effect of insoluble dietary fiber for glycemic control has been reported in different studies (De Munter *et al.*, 2007; Schulze *et al.*, 2007); however such polysaccharides have limited palatability and insoluble dietary fiber produce flatus and is not suitable for most subjects suffering from gastrointestinal disease. In addition, in prospective cohort studies, it is mainly insoluble cereal dietary fiber (i.e., cellulose and hemicelluloses) and whole grains, not soluble dietary fiber, that associated with reduced diabetes risk (De Munter *et al.*, 2007; Schulze *et al.*, 2007). In relation to consumption of non-viscous soluble palatable polysaccharides (resistant maltdextrin, RMD) a systematic review of randomized, placebo controlled trials revealed that administration of ≤ 10 g RMD per meal significantly reduces the postprandial glycemic response to a carbohydrate meal in acute studies (Livesey and Tagami, 2009), however its effect in relation to reducing risk of diabetes in long period consumption is not clear. Also RMD is fermented; it increases the production of flatus and has potential to contribute to abdominal discomfort in higher doses and continues use. (Ohkuma and Takahashi, 1990). Also RMD is more potent in drinks consumed with starch foods than when placed directly into such foods (Livesey and Tagami, 2009).

Therefore the aim of the present study was to investigate the role of low glycemic load diet having lower amount of carbohydrate and higher fat content than traditionally introduced diets as an alternative approach to reduce glycemic response to carbohydrate and also reducing HbA1c concentration of poor-controlled diabetes. We hypothesized that carbohydrate-based low glycemic load diet (GL ≈ 67), with 36% fat, and 42% carbohydrate suppress glycemic response and reduces HbA1c concentration in poor-controlled diabetes.

## 2. Materials and Methods

One hundred and twenty Poor-controlled (HbA1c > 8%) (Mahan and Escott-Stump, 2007) diabetes patients who were referred to endocrine clinic during 6 months (January 2009 to Jun 2010), and were receiving either insulin or oral medication during study were volunteers for this study. Patients were receiving conventional high carbohydrate low fat diabetes diet. Subjects were excluded if they were unwilling to consume the administrated diet and their medications have not been changed during the study. The procedures were followed in accordance with the ethical standards of the Qazvin University of Medical Science and the study was approved by the Human Research Ethics Committee of the institution. Subjects underwent on low glycemic load diet, GL = 67 (Energy = 1800 kcal, total fat = 36%, fat derived from olive oil and nuts 15%, carbohydrate = 42%, protein = 22% (Table 1) for 10 weeks. Patients were recommended to do their routine daily life style program during intervention. Fasting blood glucose (FBS), HbA1c, weight and BMI were measured before and after intervention. Data were inspected for normality of distribution before use of parametric statistics with SPSS version16 (SPSS Inc, Cary, NC). Data are reported as means ± SDs. Data were analyzed by using paired t-test and Pearson correlation to compare weight, BMI, FBS, and HbA1c of patients before and after intervention.

**Table 1:** Low glycemic load diet administrated to poor-controlled diabetes patients*

Food	Weight (g)	Protein (g)	Fat (g)	Carbohydrate (g)	GI	GL	Energy (kcal)
4 exchange from starch list, (whole-wheat bread, rice, backed beans, sliced fried potato), all low GIs	different	12	---	60	47	28	320
4 exchange from milk list (low fat milk, yogurt)	1000	32	20	48	30	14	480
8 exchange from meat and meat substitutes list (lean meat, low fat cheese, egg whites)	different	49	21	-----	---	----	440
2 exchange from vegetable list (letus, cucumber, tomato)	2 cups raw vegetable	4		10	1	1	50
4 exchange from fruit list (fresh low GI fruits, apple, orange)	480	-----		60	40	24	240
6 exchange from fat list (olive oil, olives, nuts, walnut)	different		30			----	270 (15%)
Total		97 (22%)	71 (36%)	178 (42%)		67	1800

* Source of analysis of ingredients foods: GI, & GL of foods (Taleban and Esmaeili, 1999)

## 3. Results

**Figure 1:**
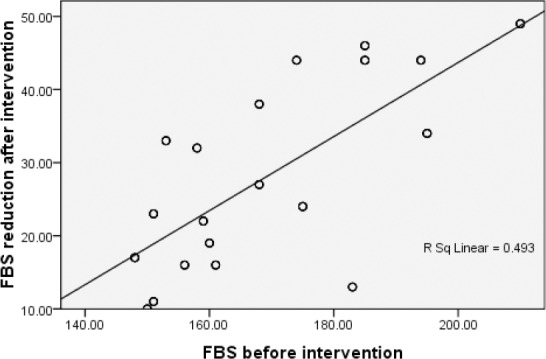
Correlation between fasting blood glucose before intervention and fasting blood glucose reduction after intervention in diabetes patients

Hundred subjects (55 M, 45 F), aged 52.8 ± 4.5 y, weight 74.0 ± 5 kg, BMI = 27.2 ± 1.9 kg/m^2^ who had recruitment criteria took part in this study. Fifteen persons had BMI ≤ 25, while 85 persons had BMI > 25. The mean values for data are shown in Table 2. FBS concentration, HbA1c percentage, weight and BMI was significantly different between two values of before and after intervention (*P* <0.001). Mean fasting blood glucose reduced by 28.1 ± 12.5 mg/dl (16.6%), HbA1c by 1.1% (11 mmol/mol) ± 0.3%, weight by 3.3 ± 1 kg and BMI by 1.2 ± 0.4 kg/m^2^ after diet intervention (*P* <0.001). There were positive weak correlation between BMI kg/m^2^ before intervention and HbA1c level reductions (*P* = 0.01, at 0.05 level, R = 0.27), between BMI kg/m^2^ reduction and HbA1c reduction (*P* = 0.01, at 0.05 level, R = 0.25), and between HbA1c concentration before intervention and HbA1c reduction (*P* < 0.001, at 0.01 level, R = 0.36). Also there was positive moderate correlation between HbA1c concentration before intervention and FBS reduction (*P* < 0.001, at 0.01 level, R = 0.52), and strong positive correlation between FBS before intervention and FBS reduction (*P* < 0.001, at 0.01 level, R = 0.70), (Table 3, Figure 1). Observed variable changes were significant in both normal and overweight groups.

**Table 2:** Blood glucose profile of diabetic patients before and after diet intervention

patients	no	age	weight	BMI	FBS	HbA1c
at baseline	100	52.8±4.5	74.0±5 CV=6.7%	27.2±1.9, CV=7%	169±17 CV=10%	8.85% (73 mmol/mol) ±0.22% CV=2%
after 10 weeks			70.7±4.6 CV=6.5%	26.0±1.8, CV=7%	141±12 CV=8%	7.81% (62 mmol/mol) ±0.27% CV=3%
*P*			*P*<0.001	*P*<0.001	*P*<0.001	P<0.001

**Table 3:** Correlation between different variables of before and after intervention

Variables	FBS reduction 28.1 ± 12.5 mg/dl	HbA1c reduction 1.1 ± 0.3
Weight 74.0±5 kg Before intervention	____________	_________
BMI 27.2±1.9 kg/m^2^ Before intervention	____________	*P* = 0.01, at 0.05 level, R= 0.27
FBS 169 ±17 mg/dl Before intervention	*P* < 0.001, at 0.01 level, R = 0.70	__________
HbA1c 8.85 ±0.22 Before intervention	*P* < 0.001, at 0.01 level, R = 0.52	*P* < 0.001, at 0.01 level, R = 0.36
Weight reduction 3.3 ± 1 kg	_____________	__________
BMI reduction 1.2 ± 0.4 kg/m^2^	_____________	*P* = 0.01, at 0.05 level, R = 0.25

## 4. Discussion

This study showed a significant effect of low glycemic load diet on FBS and HbA1c. In our study as we hypothesized, the administrated low glycemic load diet suppressed the HbA1c of poor-controlled diabetes patients to 7.8% (62 mmol/mol) ± 0.3% level which does not considered as poorly-controlled level (Mahan and Escott-Stump, 2007) and was our target in current study.

This study revealed that the more sever the dysglycemia, the greater effect of low GI diet on glycemic control was observed. This finding was parallel with point view of conducted workshop by Howlett and colleagues (Howlett and Ashwell, 2008). Similarly, researchers (Kiens and Richter, 1996) in their study found that both two isoenergetic diets which were composed of 46%, 41%, and 13% as carbohydrate, fat, and protein respectively and the carbohydrate contents were either a high GI (90) or a low GI (66), both didn’t have significant effect on normal blood glucose of healthy subjects at the end of 30 days of intervention. In addition it is reported that unavailable carbohydrate reduces fasting blood glucose or HbA1c in persons with diabetes but not in individuals having normal fasting blood glucose (Livesey *et al.*, 2008). These studies support our finding in which lower blood glucose levels and also normal blood glucose were less affected by low glycemic load diets.

Diets having composition of 50-60% of total energy as carbohydrates is recommended for diabetics and subjects with metabolic syndrome. Even recommendation of some health organizations is 55-70% carbohydrate, 15-20% proteins and 20-30% fats (Krauss *et al.*, 2000; Liu *et al.*, 2000; Franz *et al.*, 2002). However, epidemiological studies such as the Nurses Health Study and Health Professional Follow-Up Study (Hu and Willett, 2001), and also Framingham Offspring Study (Mckeown *et al.*, 2004) have demonstrated the association between glycemic load with type 2 diabetes, CVD and metabolic syndrome. High carbohydrate intake results in suboptimal glycemic control and lipoprotein profile, and subsequently increasing insulin and/or oral hypoglycemic medication requirement and weight gain (Surender and Samy, 2005), while the effect of low carbohydrate diets with 20% of total energy as carbohydrate on glycemic control was greater and independent of weight loss. However in long term compelling with restricted carbohydrate diet is difficult and adherence to such a diet having around 100 g carbohydrates a day which is far away from patients’ food habits is weak. In addition physicians are reluctant to advice such a diet to their patients. Considering accumulating evidence for benefits of restricted carbohydrate diets, the American Diabetes Association (ADA) agrees with role of carbohydrate restriction “*in weight management of type 2 diabetes, replacing carbohydrate with monounsaturated fats reduces post prandial glycemia and triglyceridemia*” and recommends that carbohydrates and monounsaturated fat together should provide 60-70% of the energy intake in which their ratio should be individualized. However, alternatively, there is statement from ADA which limits carbohydrate intake to 45-65% of the calories intake (Blades *et al.*, 1997). In our study the moderate carbohydrate diet with GL = 67g/day, including 42% carbohydrate as energy intake, and 15% of fat intake from monounsaturated fatty acids sources was almost similar to ADA’s recommendation which is more appropriate and compelling for glycemic control in long period. The GL < 80 g/day is considered low GL diet (Brand-Miller, 2005). The higher the GL, the greater the glycemic effect (Afaghi *et al.*, 2007) and insulinogenic effect (Foster-Powell *et al.*, 2002). The GL of diet in our study was 67 g/d which was even lower than maximum g/day recommendation for low GL diet.

In current study we increased the energy derived from fat up to 36%. Adherence to standard dietary advice to reduce fat intake while increasing carbohydrate intake generally increase the glycemic effect of diet. Both the quantity and quality of a carbohydrate influence postprandial glycemia, and the interaction between the two may be synergistic (Brand-Miller *et al.*, 2002). Therefore our meal plan was based on high fat foods that produce a low glycemic response (low- GI foods) and may promote weight control because they increase satiety, minimize postprandial insulin secretion, and maintain insulin sensitivity (Brand-Miller *et al.*, 2002).

Fiber consumption has significant effect on glycemic control (Howlett and Ashwell, 2008). However large amounts of fibers ingestion (25 grams per meal) is needed to achieve 10% reduction in 2 hr postprandial blood glucose level (Afaghi *et al.*, 2011). In practice due to limited palatability, produced flatus and discomfort by insoluble dietary fiber (DF), consumption of large amount of fiber is not pleasure and diabetic subjects will not compel with such a diet.

Different factors in current study may affected on glycemic control including: moderate energy intake (24 kca/per kg bod weight), low glycemic load diet, and consumption of monounsaturated fatty acids. Moderate energy intake lowers body weight (Freedman *et al.*, 2001) and consequently increases insulin sensitivity. Weight loss of 5-10% of initial body weight may significantly improve glycemic and other metabolic abnormalities, and prevents the development of diabetes in high risk populations (Tuomilehto *et al.*, 2001; Knowler *et al.*, 2002; McFarlane *et al.*, 2003). We observed 3.3%, 4.6% and 4.4% weight loss in persons having BMI ≤ 25, BMI > 25, and in total subjects respectively. Due to observed poor correlation between BMI kg/m^2^ and HbA1c and lack of any correlation between BMI reduction and FBS reduction, the weight loss in our study, less likely affected on glycemic profile improvement. We believe that the effect of administrated low glycemic load diet was dominant for weight reduction, appetite and also suppress postprandial blood glucose through slow absorption and resulting in reducing HbA1c. We did not have control group which was the limitation of our study.

## 5. Conclusion

Our provided meal plan for glycemic control of poor-controlled diabetes subjects is appropriate and further investigation for long term effect of low GI diet for glycemic control of poor-controlled diabetes patients is suggested.
